# First-in-human, phase 1 study of CM512, a TSLP/IL-13 bispecific antibody, in healthy volunteers: safety, tolerability, pharmacokinetics, pharmacodynamics, and immunogenicity

**DOI:** 10.3389/fimmu.2026.1811041

**Published:** 2026-05-28

**Authors:** Yue Cheng, Qingfeng Liu, Mengmeng Li, Yao Xie, Yuchun Men, Miao He, Huifang Wang, Xiwei Feng, Ming Zeng, Libo Zhang, Bo Chen, Jingyi Li, Ping Feng

**Affiliations:** 1 Clinical Trial Center and National Medical Products Administration Key Laboratory for Clinical Research and Evaluation of Innovative Drugs, West China Hospital of Sichuan University, Chengdu, China; 2 Department of Dermatology and Venereology, West China Hospital of Sichuan University, Chengdu, China; 3 Keymed Biosciences Co., Ltd., Chengdu, China

**Keywords:** CM512, immunogenicity, pharmacodynamics, pharmacokinetics, phase 1 study, safety

## Abstract

**Purpose:**

Type 2 chronic inflammatory diseases (e.g., atopic dermatitis and allergic rhinitis) impose a substantial global burden with unmet medical needs. Thymic stromal lymphopoietin (TSLP) and interleukin-13 (IL-13) are key pathogenic drivers, and bispecific antibodies targeting both cytokines may offer synergistic clinical benefits. CM512 is a novel recombinant bispecific antibody targeting both TSLP and IL-13 with high affinity. This first-in-human, randomized, double-blind, placebo-controlled phase 1 study evaluated the safety, tolerability, pharmacokinetics (PK), pharmacodynamics (PD), and immunogenicity of single (SAD) and multiple ascending doses (MAD) of subcutaneous CM512 in healthy adults.

**Methods:**

The SAD phase enrolled 40 healthy participants across four sequential cohorts (150, 450, 900, and 1200 mg), randomized 4:1 to receive subcutaneous CM512 or placebo per cohort. The MAD phase enrolled 24 participants across two sequential cohorts (150 and 600 mg biweekly), randomized 2:1 per cohort. The primary endpoint was safety and tolerability; secondary endpoints included PK, PD, and immunogenicity.

**Results:**

CM512 was well tolerated. Treatment-emergent adverse events (TEAEs) occurred in 71.9% (23/32) and 68.8% (11/16) of CM512 recipients in the SAD and MAD phases, with incidence comparable to placebo. All treatment-related TEAEs were mild or moderate. No serious adverse events, TEAEs leading to withdrawal, or deaths were reported. In the SAD phase, CM512 displayed linear, dose-proportional PK from 150–1200 mg, accompanied by a long terminal half-life (mean range: 58.7-73.6 days). In the MAD phase, biweekly administration resulted in accumulation (mean accumulation ratio: 2.50-3.17), consistent with its long half-life. CM512 significantly reduced free TSLP and IL-13, and sustainedly lowered blood eosinophils, IgE, and thymus activation-regulated chemokine (TARC) levels versus placebo. The incidence of treatment-emergent anti-drug antibodies in healthy participants receiving CM512 was low, at 3.1% (1/32) in the SAD phase and 0% (0/16) in the MAD phase, respectively.

**Conclusion:**

CM512 demonstrated favorable safety, linear PK, and low immunogenicity in healthy participants. It significantly reduced free target cytokines and type 2 inflammation biomarkers, supporting further clinical investigation for type 2 inflammatory diseases.

**Clinical trial registration:**

## 1 Introduction

Allergic diseases affect nearly one billion people worldwide, imposing a substantial global disease burden with unmet medical needs (1). Among these, type 2 inflammation represents a central pathogenic mechanism across the most prevalent conditions, including asthma, atopic dermatitis, and allergic rhinitis (2–4). Despite available therapies ranging from corticosteroids to biologics targeting type 2 pathways, a considerable proportion of patients still do not respond adequately. For instance, real-world evidence indicates that 45.4% of patients with moderate-to-severe atopic dermatitis do not achieve adequate response to systemic therapies, including dupilumab and traditional immunosuppressants (5). This clinical gap highlights the need for strategies that achieve broader and more comprehensive control of the type 2 inflammatory network.

Thymic stromal lymphopoietin (TSLP) and interleukin-13 (IL-13) are pivotal pathogenic drivers of these type 2 inflammatory diseases (2). TSLP is an upstream epithelial cell-derived alarmin cytokine that promotes Th2 cell differentiation and Th2 cytokine-associated inflammation (6), with increased expression detected across multiple type 2 inflammatory diseases (7–10). IL-13 is a downstream Th2 cell-derived effector cytokine secreted at elevated levels in allergic tissues and plays a central regulatory role in type 2 immune response (2, 11, 12). Monoclonal antibodies targeting these cytokines have demonstrated safety and efficacy in clinical trials and are approved for the treatment of asthma and atopic dermatitis (13–18). It is hypothesized that bispecific anti-TSLP/IL-13 antibodies may offer enhanced clinical benefits through synergistic inhibition of both pathways (19, 20). By concurrently blocking TSLP and IL-13, this dual mechanism combines upstream alarmin blockade with downstream effector inhibition to achieve more comprehensive control of the complex inflammatory network in type 2 inflammation. Currently, bispecific anti-TSLP/IL-13 antibodies remain in development, with two agents, lunsekimig and CM512, under clinical investigation. Positive phase 1 results with lunsekimig further support the potential of this dual inhibition strategy in biological therapeutics (20).

CM512 is a novel recombinant bispecific antibody that targets both TSLP and IL-13 with high affinity. Unlike lunsekimig, which is a Nanobody^®^-based molecule, CM512 is a full-length IgG1 bispecific antibody, thereby expanding the structural diversity of therapeutic options within this emerging class (20, 21). By simultaneously blocking the binding of TSLP to its receptor and the IL-13/IL-13Rα1 complex to IL-4Rα, CM512 achieved synergistic inhibition of downstream signaling activation, leading to suppressed pathogenic cell proliferation and reduced release of proinflammatory cytokines. CM512 exhibited an extended half-life, low immunogenicity, and no apparent toxicity in preclinical studies (21). These properties position CM512 as a promising therapeutic candidate for type 2 inflammatory diseases.

Based on these encouraging preliminary findings, we conducted a first-in-human, dose-escalation phase 1 trial (NCT06553209). This trial was designed to evaluate the safety, tolerability, pharmacokinetics (PK), pharmacodynamics (PD), and immunogenicity of CM512 in both healthy adults and patients with moderate-to-severe atopic dermatitis. Herein, we present the initial results from healthy cohorts with single (SAD) and multiple ascending doses (MAD) of subcutaneous CM512. Data from atopic dermatitis cohorts will be analyzed and published separately.

## 2 Methods

### 2.1 Study design and participants

**Figure 1:**
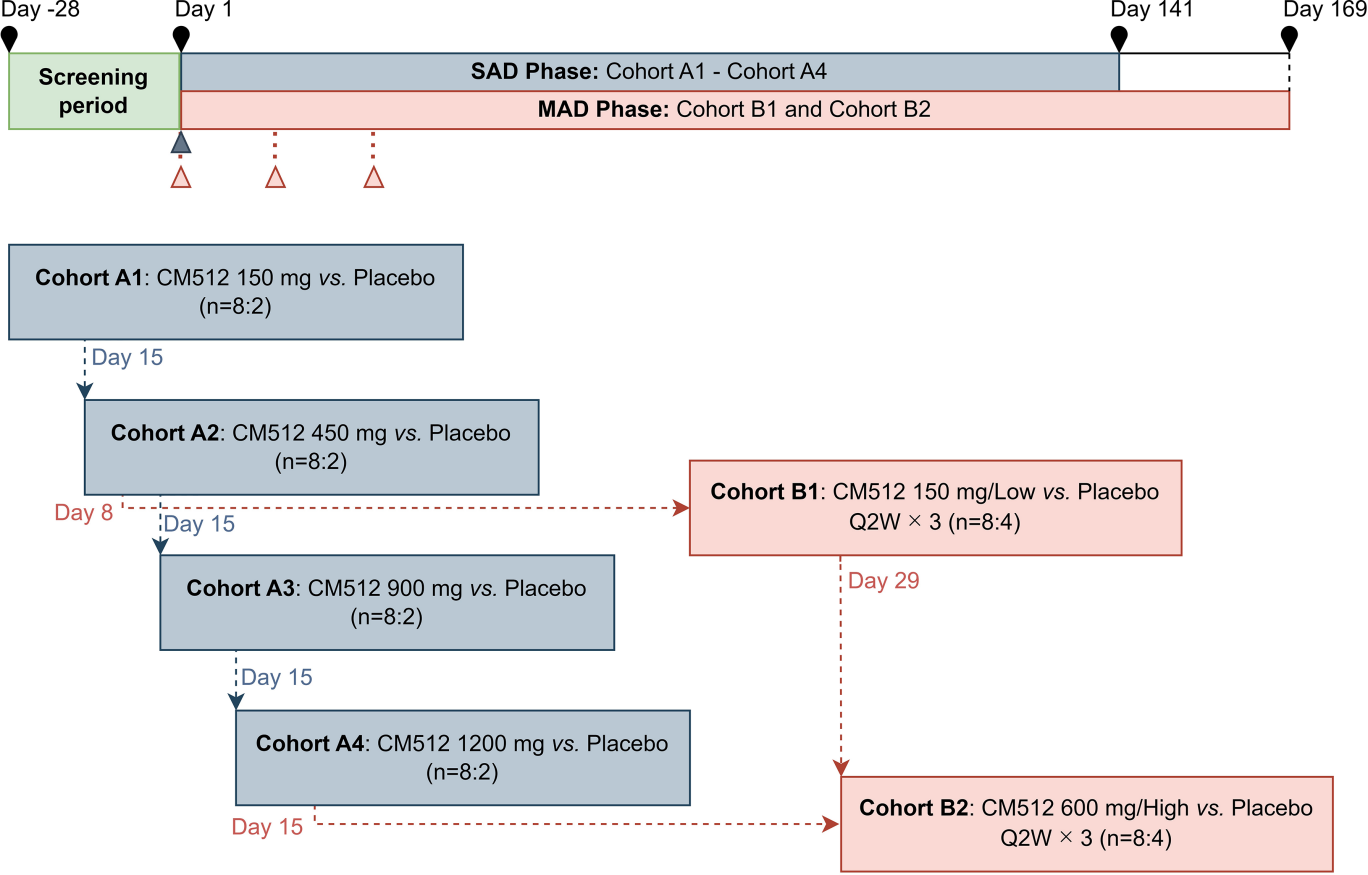
Schematic of study design. Solid triangles indicate dosing time points. In the SAD phase, a single dose of CM512 was administered subcutaneously on Day 1. In the MAD phase, CM512 was administered subcutaneously Q2W, with three doses given on Days 1, 15, and 29. MAD, multiple ascending dose; Q2W, every two weeks; SAD, single ascending dose.

This was a first-in-human, randomized, double-blind, placebo-controlled phase 1 trial of CM512 in healthy participants in China. The primary objective was to evaluate the safety and tolerability of CM512 following both SAD and MAD regimens, with PK, PD, and immunogenicity as secondary objectives. The study design and dose escalation process are illustrated in **Figure 1**. This study enrolled healthy volunteers aged 18 to 45 years, with a body mass index (BMI) between 18 and 26 kg/m^2^ and in good general health. Key exclusion criteria included any clinically significant abnormalities in medical history, physical examination, vital signs, laboratory tests, electrocardiogram (ECG), or chest imaging; known hypersensitivity to anti-IL-13 or anti-TSLP antibodies or history of severe drug or food allergy; and recent infection, trauma, or surgery. Full eligibility criteria are detailed in the **Supplementary Methods**.

The study comprised SAD and MAD parts. In the SAD phase, a total of 40 participants were sequentially enrolled into four cohorts receiving ascending dose levels of 150 mg, 450 mg, 900 mg, and 1200 mg (the rationale for dose selection is provided in the **Supplementary Methods**). Within each cohort, eligible participants were randomized in a 4:1 ratio to receive a single subcutaneous dose of either CM512 or placebo on Day 1. Dose escalation to the next cohort proceeded only after the previous dose level was deemed safe based on the safety assessment on Day 15.

The MAD phase included a total of 24 participants across two sequential cohorts to evaluate multiple subcutaneous doses of CM512 at 150 mg (low dose) and 600 mg (high dose) every two weeks (Q2W). Healthy participants were randomized 2:1 within each cohort to receive CM512 or placebo Q2W for a total of three doses, administered on Days 1, 15, and 29. Initiation of the MAD low-dose cohort was based on the established safety of the SAD 450 mg cohort on the Day 8 safety assessment. Dose escalation to the high-dose cohort required both the Day 29 safety assessment of the MAD low-dose cohort and the Day 15 assessment of the final SAD cohort.

The study protocol was reviewed and approved by the Ethics Committee on Clinical Trial, West China Hospital of Sichuan University (Approval No.: HX-1RB-AF.14-V5.0). This study was conducted in strict compliance with the trial protocol, the Declaration of Helsinki, Good Clinical Practice, and all applicable laws and guidelines. Written informed consent was obtained from all participants before their participation.

### 2.2 Safety, tolerability, pharmacokinetics, pharmacodynamics, and immunogenicity analysis of CM512

The safety and tolerability of CM512 were assessed as primary objectives. Safety and tolerability were monitored up to Day 141 (± 7 days) in the SAD phase and Day 169 (± 7 days) in the MAD phase, as assessed by treatment-emergent adverse events (TEAEs) and clinically significant abnormalities in physical examinations, vital signs, 12-lead ECG, laboratory tests, and pregnancy tests. TEAEs were recorded throughout the trial, including their severity, resolution, and relationship to study drug. Laboratory tests included complete blood count, blood biochemistry, coagulation, and urinalysis.

PK, PD, and immunogenicity were assessed as secondary objectives. PK parameters following a single dose of CM512 in the SAD phase, as well as after the first and last doses in the MAD phase were assessed, including maximum observed concentration (C_max_), time to C_max_ (T_max_), area under the concentration-time curve from time zero to the last quantifiable concentration (AUC_0-t_), area under the concentration-time curve from time zero extrapolated to infinity (AUC_inf_), area under the concentration-time curve over a dosing interval (AUC_tau_), terminal half-life (t_1/2z_), apparent total body clearance (CL/F), apparent volume of distribution (V_z_/F), mean residence time from zero to the last quantifiable concentration (MRT_0-t_) and to infinity (MRT_inf_), and the accumulation ratios (R_ac_) of the last dose to the first dose for C_max_ (R_ac__C_max_) and AUC_tau_ (R_ac__AUC_tau_). PD effects were assessed by percent change from baseline in blood eosinophil (EOS) counts, serum total immunoglobulin E (IgE), and serum thymus activation-regulated chemokine (TARC), along with exploratory biomarkers of free TSLP and IL-13. Immunogenicity was evaluated based on the incidence of anti-drug antibodies (ADA).

### 2.3 Biospecimen collection and analysis

In the SAD and MAD phases, peripheral blood samples for PK, PD, and immunogenicity analysis were collected predose and at prespecified time points after dosing. The serum concentration of CM512 was measured using a validated enzyme-linked immunosorbent assay (ELISA) with a lower limit of quantification (LLOQ) of 40.0 ng/mL. Serum total IgE was quantified using a validated electrochemiluminescence immunoassay (ECLIA) with a LLOQ of 3.00 ng/mL. Serum TARC levels were determined using a validated Quantikine ELISA Human CCL17/TARC immunoassay (R&D Systems Inc, USA) with a LLOQ of 31.25 pg/mL. Serum free TSLP was quantified using a validated electrochemiluminescence assay (ECLA) with a LLOQ of 240 fg/mL. Serum free IL-13 was quantified using a validated ECLIA method with a limit of detection (LOD) of 14.7 fg/mL. Anti-drug antibodies (ADAs) against CM512 were detected using a bridging-ELISA method, with a minimal required dilution of 1:95.

### 2.4 Statistical analysis

The sample size was not determined based on statistical considerations. A total of 64 healthy participants were planned for enrollment, with 10 in each of the four SAD cohorts and 12 in each of the two MAD cohorts.

Continuous variables were summarized as means ± standard deviations (SD) for normally distributed data and as median (interquartile range, IQR) for non-normally distributed/skewed data. Categorical variables were presented as counts and percentages. All statistical analyses were performed using SAS software version 9.4 (SAS Institute, Cary, NC, USA). All analyses in this study were based on observed values, and missing data were not imputed. Baseline characteristics and participant disposition were analyzed descriptively.

Demographic data were analyzed based on the full analysis set, including all randomized participants with at least one dose of CM512 or placebo. Safety analyses were conducted in the safety set, which included all participants who received at least one dose of CM512 or placebo. All TEAEs were coded according to the Medical Dictionary for Regulatory Activities (MedDRA) version 28.1 using system organ class (SOC) and preferred terms (PT). The severity of TEAEs was graded according to Common Terminology Criteria for Adverse Events (CTCAE) version 5.0. The frequency and incidence of TEAEs were summarized descriptively for all SAD and MAD cohorts.

PK analyses were performed in the PK concentration set and PK parameter set. Specifically, the PK concentration set included all participants who received at least one dose of CM512 or placebo and had at least one valid PK concentration measurement after dosing, while the PK parameter set comprised all such dosed participants with at least one valid PK parameter measurement after dosing. Semi-log-transformed serum concentration-time profiles following subcutaneous CM512 administration were plotted for each SAD and MAD cohort. PK parameters were calculated using Phoenix WinNonlin version 8.4 (Certara, Radnor, PA, USA) with a noncompartmental analysis approach. PK parameters were summarized descriptively as means ± SDs, except for T_max_, which was presented as median (minimum, maximum). Furthermore, dose proportionality of CM512 PK parameters was assessed using a power model in the SAD phase.

PD analyses were performed in the PD set, which included all participants who received at least one dose of CM512 or placebo and had at least one valid PD measurement. Absolute values and percent changes from baseline in blood EOS, serum total IgE, TARC, TSLP, and IL-13 were analyzed descriptively by study visit using median (IQR). Values below the quantitation limit were imputed using the respective limit value for each analyte: specifically, the LLOQ for TSLP and the LOD for IL-13.

Immunogenicity analyses were conducted in the immunogenicity set, which included all participants who received at least one dose of CM512 or placebo and had at least one valid immunogenicity measurement. The incidence and titer ranges for pre-existing ADAs, treatment-emergent ADAs, treatment-boosted ADAs were summarized descriptively.

## 3 Results

### 3.1 Participants

Between August and October 2024, 40 healthy volunteers were enrolled in the SAD phase and randomized to receive placebo (*n* = 8) or CM512 at 150 mg (*n* = 8), 450 mg (*n* = 8), 900 mg (*n* = 8), or 1200 mg (*n* = 8). One participant in the 900 mg group discontinued early at the participant’s request; All others completed the study. All participants were included in safety, PK, PD, and immunogenicity analyses, and 32 CM512-treated participants contributed to PK parameter analyses (**Supplementary Figure 1**). In the SAD phase, 62.5% (25/40) were male, the mean age was 26.6 ± 4.3 years, 92.5% (37/40) were Han Chinese, and the mean BMI was 21.8 ± 2.0 kg/m² (**Table 1**).

**Table 1:** Demographic characteristics of participants in the SAD and MAD phases.

Characteristics	SAD phase						MAD phase			
	Pooled placebo (*n* = 8)	CM512 150 mg (*n* = 8)	CM512 450 mg (*n* = 8)	CM512 900 mg (*n* = 8)	CM512 1200 mg (*n* = 8)	Total (*n* = 40)	Pooled placebo (*n* = 8)	150mg Q2W (*n* = 8)	600mg Q2W (*n* = 8)	Total (*n* = 24)
Age, years	27.3 ± 4.9	26.5 ± 4.3	24.4 ± 4.6	27.9 ± 4.9	26.8 ± 2.9	26.6 ± 4.3	25.8 ± 2.8	27.4 ± 6.0	27.0 ± 5.0	26.7 ± 4.6
Sex, n (%)										
Male	7 (87.5)	5 (62.5)	5 (62.5)	6 (75.0)	2 (25.0)	25 (62.5)	7 (87.5)	5 (62.5)	6 (75.0)	18 (75.0)
Female	1 (12.5)	3 (37.5)	3 (37.5)	2 (25.0)	6 (75.0)	15 (37.5)	1 (12.5)	3 (37.5)	2 (25.0)	6 (25.0)
Ethnicity, n (%)										
Han	8 (100)	8 (100)	6 (75.0)	7 (87.5)	8 (100)	37 (92.5)	8 (100)	8 (100)	8 (100)	24 (100)
Others	0	0	2 (25.0)	1 (12.5)	0	3 (7.5)	0	0	0	0
Weight, kg	60.0 ± 6.4	56.4 ± 5.2	61.8 ± 8.4	60.9 ± 7.6	58.3 ± 8.7	59.5 ± 7.3	60.4 ± 10.3	57.0 ± 5.7	58.0 ± 4.7	58.4 ± 7.2
BMI, kg/m^2^	22.2 ± 2.3	20.6 ± 1.3	21.9 ± 1.6	22.5 ± 2.0	21.9 ± 2.6	21.8 ± 2.0	21.5 ± 2.4	21.6 ± 2.2	21.6 ± 1.7	21.6 ± 2.0

Continuous data are presented as mean ± SD; Categorical data are presented as number (percentage). BMI, body mass index; MAD, multiple ascending dose; Q2W, every two weeks; SAD, single ascending dose.

Between October and November 2024, 24 healthy volunteers were enrolled in the MAD phase and randomized to placebo (*n* = 8), CM512 at 150 mg (*n* = 8), or CM512 at 600 mg (*n* = 8). All participants completed the study and were included in the relevant analyses; 16 CM512-treated participants contributed to PK parameter analyses. Baseline characteristics were similar to those in the SAD phase: 75% (18/24) were male, the mean age was 26.7 ± 4.6 years, all were Han Chinese, and the mean BMI was 21.6 ± 2.0 kg/m² (**Table 1**).

Overall, baseline characteristics were well-balanced across placebo and CM512 dose groups in both the SAD and MAD phases.

### 3.2 Safety and tolerability

In the SAD phase, TEAEs occurred in 71.9% (23/32) of the pooled CM512 group and 87.5% (7/8) of the placebo group. Treatment-related TEAEs were reported in 40.6% (13/32) of the pooled CM512 group and 50.0% (4/8) of the placebo group, respectively. All treatment-related TEAEs were of Grade 1 or 2 in severity. The most frequent TEAEs were hypertriglyceridaemia (CM512: 9/32, 28.1%; Placebo: 1/8, 12.5%), alanine aminotransferase increased (CM512: 5/32, 15.6%; Placebo: 1/8, 12.5%), white blood cells urine positive (CM512: 3/32, 9.4%; Placebo: 0/8, 0%), aspartate aminotransferase increased (CM512: 3/32, 9.4%; Placebo: 0/8, 0%), and upper respiratory tract infection (CM512: 3/32, 9.4%; Placebo: 0/8, 0%).

In the MAD phase, 68.8% (11/16) of the pooled CM512 group and 50.0% (4/8) of the placebo group experienced TEAEs. Treatment-related TEAEs occurred in 50.0% (8/16) and 37.5% (3/8), respectively, all of which were Grade 1 or 2. The most common TEAE was hypertriglyceridaemia (CM512: 4/16, 25.0%; Placebo: 2/8, 25.0%).

Across both the SAD and MAD phases, no SAEs, TEAEs leading to early withdrawal, or deaths were reported. No apparent dose-dependent trends in TEAEs incidence were identified. No consistent clinically significant abnormalities were observed in physical examinations, vital signs, 12-lead ECG, or routine laboratory tests. A complete summary of TEAEs is provided in **Table 2**; **Supplementary Tables S1**, **S2**.

**Table 2:** Overview of adverse events.

*n* (%)	SAD phase						MAD phase			
	Pooled placebo (*n* = 8)	Pooled CM512 (*n* = 32)	CM512 150 mg (*n* = 8)	CM512 450 mg (*n* = 8)	CM512 900 mg (*n* = 8)	CM512 1200 mg (*n* = 8)	Pooled placebo (*n* = 8)	Pooled CM512 (*n* = 16)	150mg Q2W (*n* = 8)	600mg Q2W (*n* = 8)
TEAEs	7 (87.5)	23 (71.9)	4 (50.0)	7 (87.5)	8 (100)	4 (50.0)	4 (50.0)	11 (68.8)	7 (87.5)	4 (50.0)
Grade 1	5 (62.5)	22 (68.8)	4 (50.0)	7 (87.5)	7 (87.5)	4 (50.0)	3 (37.5)	9 (56.3)	6 (75.0)	3 (37.5)
Grade 2	3 (37.5)	7 (21.9)	0	2 (25.0)	4 (50.0)	1 (12.5)	3 (37.5)	5 (31.3)	3 (37.5)	2 (25.0)
Grade ≥3	0	1 (3.1)	0	1 (12.5)	0	0	1 (12.5)	1 (6.3)	1 (12.5)	0
Treatment-related TEAEs	4 (50.0)	13 (40.6)	1 (12.5)	3 (37.5)	7 (87.5)	2 (25.0)	3 (37.5)	8 (50.0)	5 (62.5)	3 (37.5)
Grade 1	3 (37.5)	12 (37.5)	1 (12.5)	3 (37.5)	6 (75.0)	2 (25.0)	3 (37.5)	6 (37.5)	4 (50.0)	2 (25.0)
Grade 2	1 (12.5)	2 (6.3)	0	0	2 (25.0)	0	1 (12.5)	4 (25.0)	2 (25.0)	2 (25.0)
Grade ≥3	0	0	0	0	0	0	0	0	0	0
SAEs	0	0	0	0	0	0	0	0	0	0
TEAEs leading to early withdrawal	0	0	0	0	0	0	0	0	0	0
TEAEs leading to death	0	0	0	0	0	0	0	0	0	0
Most common TEAEs*										
Hypertriglyceridaemia	1 (12.5)	9 (28.1)	3 (37.5)	1 (12.5)	4 (50.0)	1 (12.5)	2 (25.0)	4 (25.0)	2 (25.0)	2 (25.0)
Alanine aminotransferase increased	1 (12.5)	5 (15.6)	0	3 (37.5)	2 (25.0)	0	1 (12.5)	2 (12.5)	1 (12.5)	1 (12.5)
White blood cells urine positive	0	3 (9.4)	0	1 (12.5)	1 (12.5)	1 (12.5)	0	1 (6.3)	1 (12.5)	0
Aspartate aminotransferase increased	0	3 (9.4)	0	1 (12.5)	1 (12.5)	1 (12.5)	2 (25.0)	0	0	0
Upper respiratory tract infection	0	3 (9.4)	0	0	2 (25.0)	1 (12.5)	1 (12.5)	2 (12.5)	2 (25.0)	0

n (%), number and percentage of participants with at least one TEAE. MAD, multiple ascending dose; Q2W, every two weeks; SAD, single ascending dose; SAEs, serious adverse events; TEAEs, treatment emergent adverse events, defined as any adverse events that occur or worsen relative to baseline after the first dose of the study drug.

*The most common TEAEs were defined as those that occurred in ≥3 participants in either pooled placebo or pooled CM512 groups during the SAD or MAD phases.

### 3.3 Pharmacokinetics

**Figure 2:**
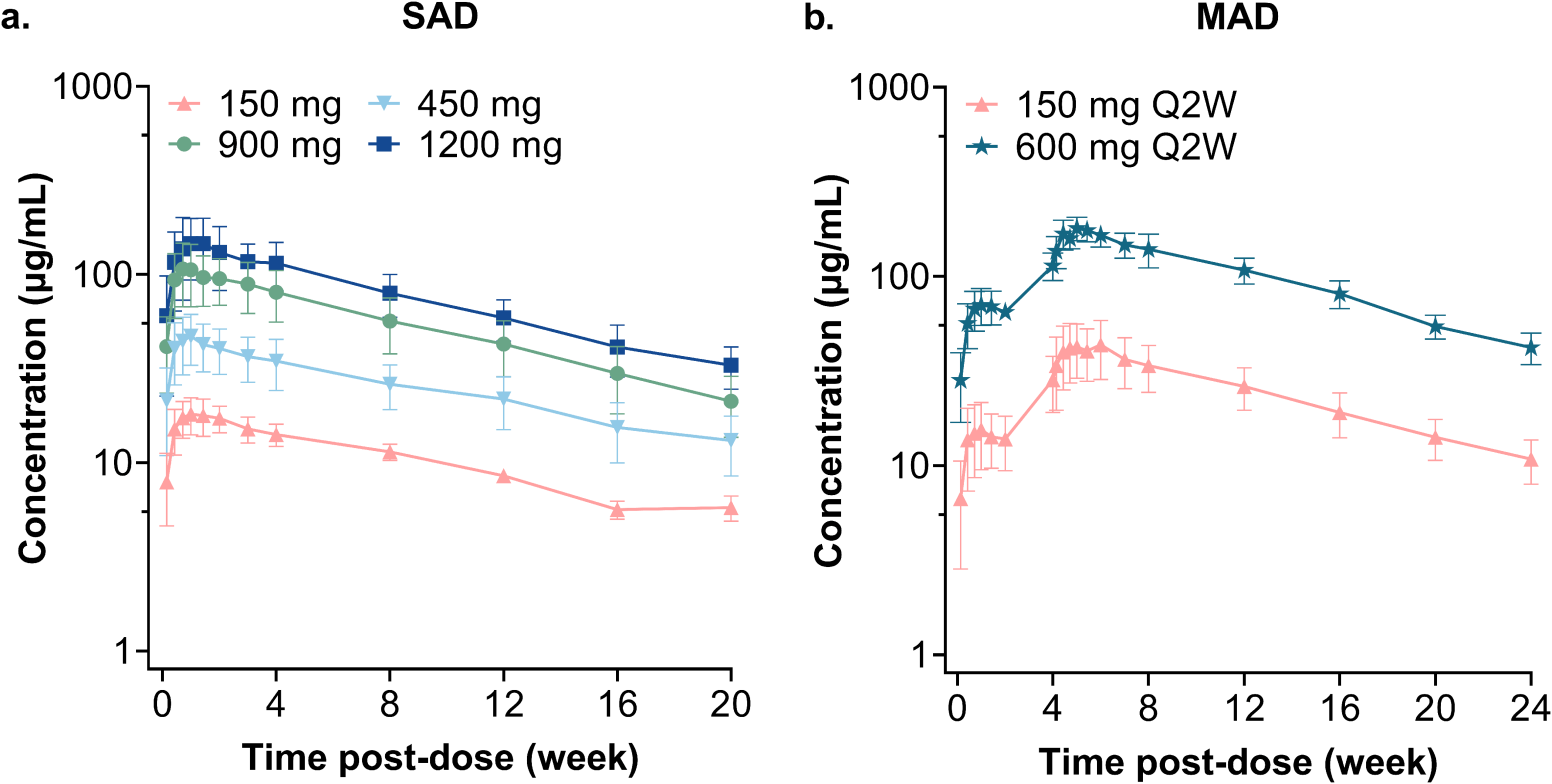
Mean serum concentration-time profiles of CM512 in healthy participants. **(A)** Following a single subcutaneous administration of ascending doses (150, 450, 900, and 1200 mg) in the SAD phase; **(B)** Following multiple subcutaneous administration of ascending doses (150 and 600 mg) every two weeks in the MAD phase. Data are presented on a semi−logarithmic scale. Error bars represent standard deviation. MAD, multiple ascending dose; Q2W, every two weeks; SAD, single ascending dose.

In the SAD phase, single subcutaneous doses of CM512 from 150 to 1200 mg resulted in rapid serum concentrations elevation and subsequent peak attainment (**Figure 2A**). The median T_max_ values were comparable across dose groups, ranging from 144 to 204 hours postdose. CM512 exposure increased in a dose-dependent manner, with mean C_max_ ranging from 19.1 ± 3.52 μg/mL at the 150 mg dose to 153 ± 54.5 μg/mL at the 1200 mg dose. Corresponding values for AUC_0-t_ were 1410 ± 133 to 10400 ± 2940 day·μg/mL, and for AUC_inf_ were 2040 ± 201 to 13300 ± 3530 day·μg/mL. Elimination parameters were consistent across dose groups, indicating linear elimination. The t_1/2z_ ranged from 58.7 ± 10.3 to 73.6 ± 13.6 days. The corresponding PK parameters in the SAD phase are shown in **Table 3**. Furthermore, dose proportionality analysis revealed that C_max_, AUC_0-t_, and AUC_inf_ increased proportionally within dose range of 150 to 1200 mg, with slope estimates approximating 1 (range: 0.88-0.98) for each PK parameter (**Supplementary Figure S2**).

**Table 3:** Pharmacokinetic parameters following a single subcutaneous dose of CM512 in the SAD phase.

PK parameters	CM512–150 mg^a^ (*n* = 8)	CM512–450 mg (*n* = 8)	CM512–900 mg^b^ (*n* = 8)	CM512–1200 mg (*n* = 8)
T_max_, h	204 (168, 336)	168 (120, 240)	144 (72, 385)	167 (119, 505)
C_max_, μg/mL	19.1 ± 3.52	48.2 ± 14.8	112 ± 37.9	153 ± 54.5
AUC_0-t_, day·μg/mL	1410 ± 133	3510 ± 1010	7520 ± 2260	10400 ± 2940
AUC_inf_, day·μg/mL	2040 ± 201	4900 ± 1590	9350 ± 2930	13300 ± 3530
t_1/2z_, day	73.6 ± 13.6	72.4 ± 11.7	58.7 ± 10.3	61.2 ± 6.47
V_z_/F, L	7.83 ± 1.22	10.7 ± 5.11	8.90 ± 3.40	8.53 ± 2.76
CL/F, mL/h	3.10 ± 0.320	4.34 ± 1.95	4.40 ± 1.49	3.99 ± 1.07
MRT_0-t_, day	55.7 ± 2.69	56.37 ± 1.99	52.38 ± 1.52	53.20 ± 3.49
MRT_inf_, day	112 ± 20.8	110 ± 14.1	86.3 ± 12.6	91.7 ± 11.4

T_max_ is presented as median (minimum, maximum); Other PK parameters are presented as mean ± SD. AUC_0-t_ was determined over 140 days post-dose in the SAD phase. AUC_0-t_, area under the concentration-time curve from time zero to the last quantifiable concentration; AUC_inf_, area under the concentration-time curve from time zero extrapolated to infinity; CL/F, apparent total body clearance; C_max_, maximum observed concentration; MRT_0-t_, mean residence time from zero to the last quantifiable concentration; MRT_inf_, mean residence time from zero extrapolated to infinity; PK, pharmacokinetics; SAD, single ascending dose; t_1/2z_, terminal half-life; T_max_, time to maximum observed concentration; V_z_/F, apparent volume of distribution.

^a^ For one subject in the 150 mg group (Adj R² <0.85), only C_max_, T_max_, AUC_0-t_, and MRT_0-t_ were included in the descriptive statistics.

^b^ One subject in the 900 mg group withdrew early, only C_max_, T_max_, AUC_0-t_, and MRT_0-t_ were included in the descriptive statistics.

In the MAD phase, escalating serum concentrations were observed with repeated dosing (**Figure 2B**). Following the first subcutaneous administration of CM512 at 150 mg or 600 mg, the median T_max_ were 168 and 241 hours postdose, respectively. After the third dose, the median T_max_ were 277 and 168 hours postdose for the two dose groups, respectively. Similar to the SAD phase, the drug exposure of the two dose groups in the MAD phase increased with dose. Both C_max_ and AUC_0-t_ after the last dose were higher than after the first dose. Elimination parameters were similar between the two dose groups, with t_1/2z_ of 68.3 ± 6.08 and 58.1 ± 7.81 days, respectively. After the last dose, the R_ac__C_max_ were 2.97 ± 0.466 and 2.50 ± 0.241, and R_ac__AUC_tau_ were 3.17 ± 0.497 and 2.79 ± 0.347, indicating moderate accumulation following three consecutive biweekly doses of 150 mg or 600 mg CM512. The corresponding PK parameters in the MAD phase are shown in **Table 4**.

**Table 4:** Pharmacokinetic parameters following multiple subcutaneous doses of CM512 in the MAD phase.

PK parameters	First dose		Last dose	
	150 mg (*n* = 8)	600 mg (*n* = 8)	150 mg (*n* = 8)	600 mg (*n* = 8)
T_max_, h	168 (120, 336)	241 (120, 336)	277 (72.2, 314)	168 (72.0, 241)
C_max_, μg/mL	15.9 ± 6.06	76.0 ± 13.0	45.9 ± 15.4	189 ± 25.8
AUC_0-t_, day·μg/mL	183 ± 69.4	838 ± 161	3360 ± 911	13400 ± 1960
AUC_inf_, day·μg/mL	/	/	4420 ± 1170	17000 ± 2670
AUC_tau_, day·μg/mL	/	/	564 ± 187	2300 ± 286
t_1/2z_, day	/	/	68.3 ± 6.08	58.1 ± 7.81
V_z_/F, L	/	/	30.0 ± 13.8	22.2 ± 4.53
CL/F, mL/h	/	/	12.7 ± 5.62	11.0 ± 1.59
MRT_inf_, day	/	/	106 ± 13.0	96.4 ± 8.11
R_ac__C_max_	/	/	2.97 ± 0.466	2.50 ± 0.241
R_ac__AUC_tau_	/	/	3.17 ± 0.497	2.79 ± 0.347

T_max_ is presented as median (minimum, maximum); Other PK parameters are presented as mean ± SD. In the MAD phase, AUC_0-t_ was assessed across two intervals: 14 days after the first dose (D1-D15) and 140 days after the last dose (D29-end of study). R_ac_ was defined as the accumulation ratio of the last dose relative to the first dose. AUC_0-t_, area under the concentration-time curve from time zero to the last quantifiable concentration; AUC_inf_, area under the concentration-time curve from time zero extrapolated to infinity; AUC_tau_, area under the concentration-time curve over a dosing interval; CL/F, apparent total body clearance; C_max_, maximum observed concentration; MAD, multiple ascending dose; MRT_inf_, mean residence time from zero extrapolated to infinity; PK, pharmacokinetics; R_ac__C_max_, accumulation ratio of maximum observed concentration; R_ac__AUC_tau_, accumulation ratio of the area under the curve over a dosing interval; t_1/2z_, terminal half-life; T_max_, time to maximum observed concentration; V_z_/F, apparent volume of distribution.

### 3.4 Pharmacodynamics

**Figure 3:**
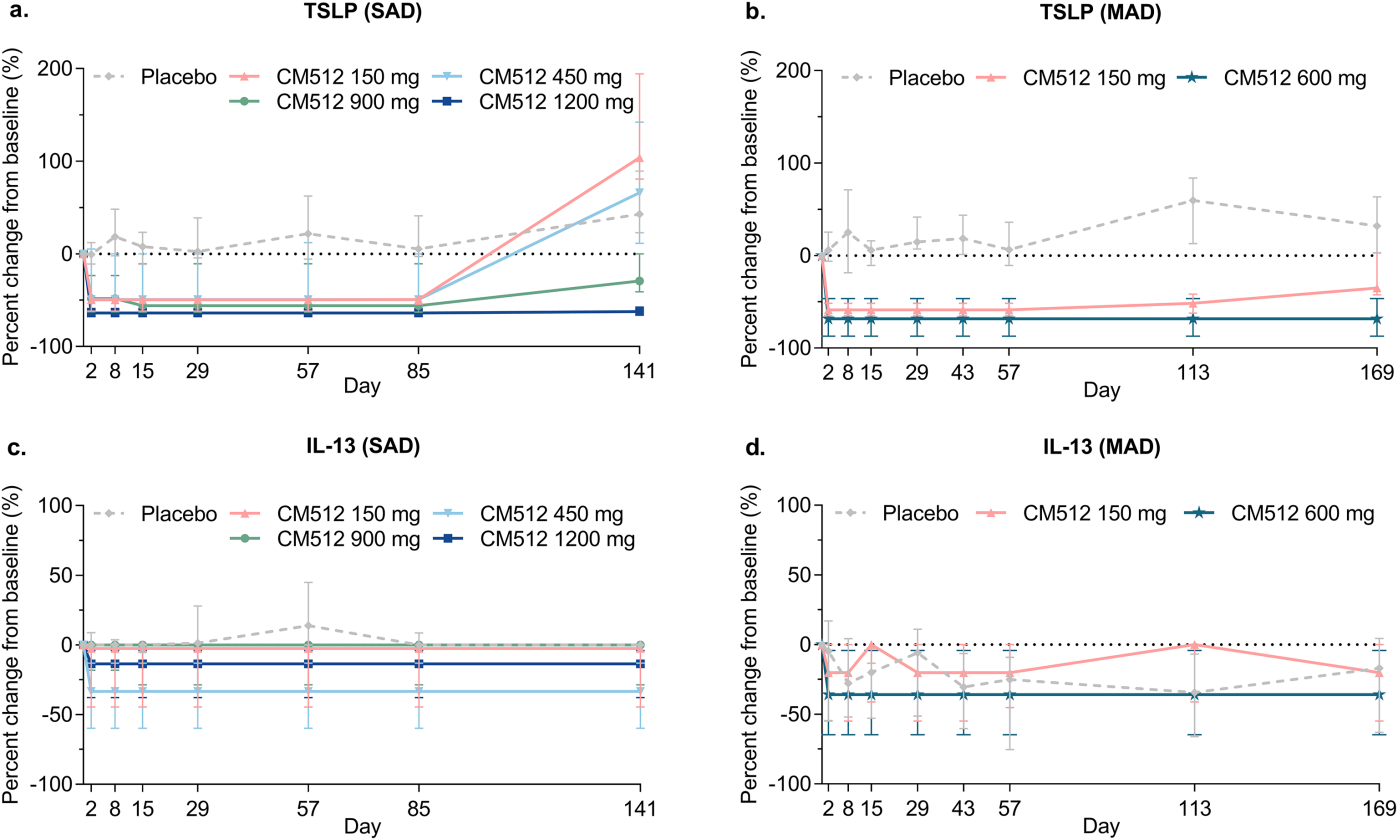
Median percent changes from baseline in serum concentrations of free TSLP and IL-13. Data points represent the median percent changes from baseline in TSLP **(A, B)** and IL-13 **(C, D)** in the SAD and MAD phases. Error bars indicate the interquartile range. For TSLP, concentrations below the LLOQ were assigned the LLOQ value. For IL-13, concentrations below the LOD were assigned the LOD value. IL-13, interleukin-13; LLOQ, lower limit of quantification; LOD, limit of detection; MAD, multiple ascending dose; SAD, single ascending dose; TSLP, thymic stromal lymphopoietin.

CM512 administration induced pronounced reductions in the target biomarkers, free IL-13 and TSLP, in both the SAD and MAD phases, confirming effective target engagement. Decreases were observed within 2 days post−dose across all CM512 dose groups in both phases, with median concentrations falling to the respective assay limit levels. These levels were maintained until the penultimate visit (Day 85 for SAD, Day 113 for MAD). At the end-of-study visit, TSLP levels showed recovery in the 150 mg, 450 mg, and 900 mg groups of the SAD phase and the 150 mg group of the MAD phase, while the 1200 mg group of the SAD phase and the 600 mg group of the MAD phase remained at the LLOQ. For IL-13, median concentrations remained at the LOD level through the end-of-study visit (Day 141 for SAD, Day 169 for MAD) across all dose groups. (**Figures 3A–D**).

**Figure 4:**
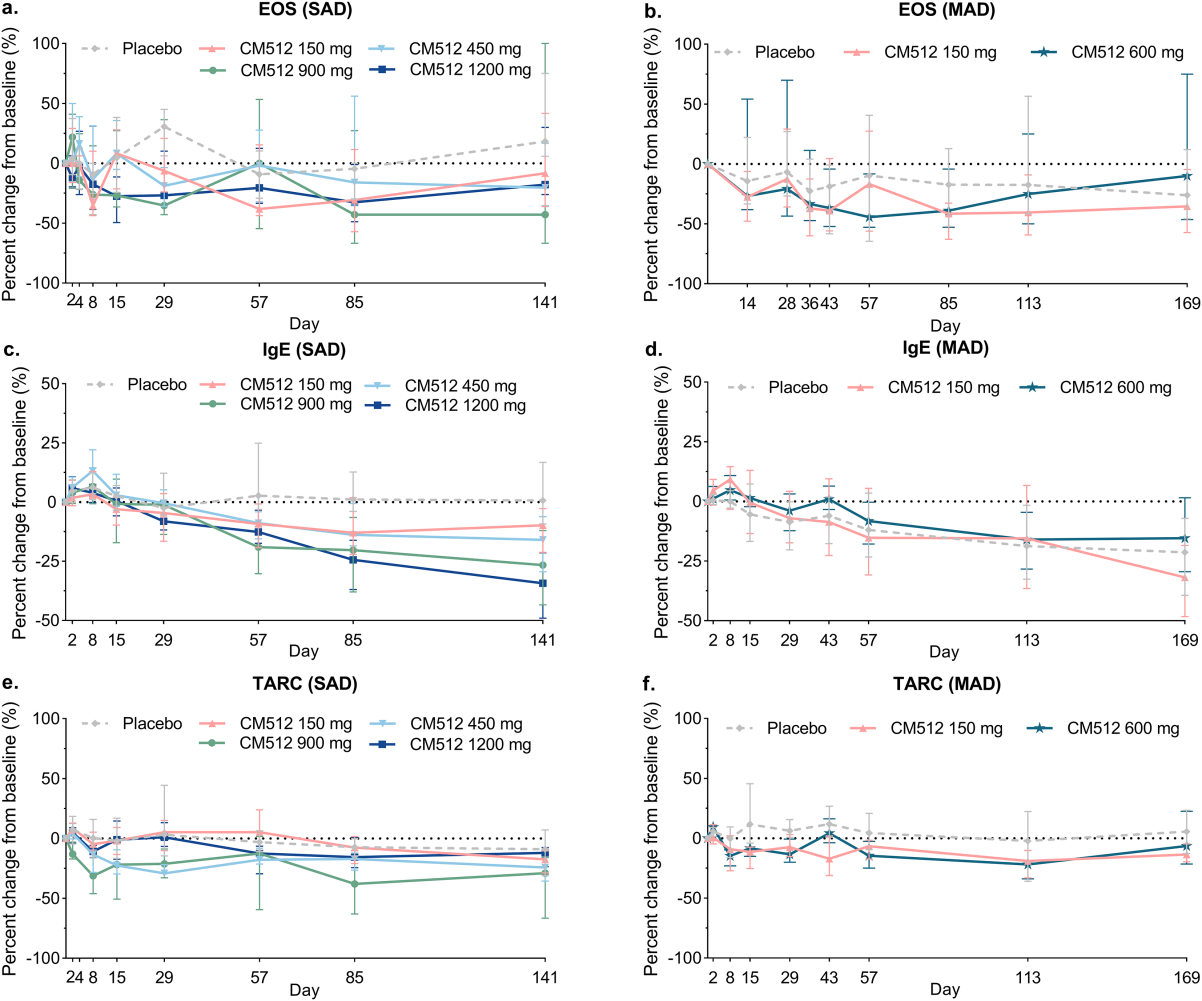
Median percent changes from baseline in pharmacodynamic markers. Median percent changes from baseline in EOS **(A, B)**, IgE **(C, D)**, and TARC **(E, F)** are shown for the SAD and MAD phases. Error bars indicate the interquartile range. EOS, eosinophil; IgE, immunoglobulin E; MAD, multiple ascending dose; SAD, single ascending dose; TARC, thymus activation-regulated chemokine.

Reductions in EOS, IgE, and TARC were also observed across CM512 dose groups in both the SAD and MAD phases (**Figure 4**). In the SAD phase, these biomarkers showed rapid and sustained decreases following CM512 administration. The most pronounced reductions in EOS and TARC occurred in the 900 mg group, with median decreases maintained through Day 141. For EOS, the median percent change from baseline reached its nadir at Day 85 and Day 141, with values of -42.9% (IQR: -66.7% to 27.3%) and -42.9% (IQR: -66.7% to 100.0%), respectively. The reduction in TARC was most marked at Day 85, with a median percent change from baseline of -37.9% (IQR: -63.0% to -10.1%) (**Figures 4A, E**). In contrast, the most pronounced and sustained reduction in IgE was observed in the 1200 mg group, with a median percent change from baseline of -34.4% (IQR: -49.0% to -21.4%) through Day 141 (**Figure 4C**). Other CM512 dose groups also showed numerically greater decreases in these biomarkers relative to placebo. In the MAD phase, reductions in EOS, IgE, and TARC were also observed across CM512 groups (**Figures 4B, D, F**). Across both the SAD and MAD phases, no evident dose-dependent pattern was observed in the magnitude of biomarker decreases.

### 3.5 Immunogenicity

In the SAD phase, treatment-emergent ADAs were detected in 1 of 32 participants (3.1%) receiving CM512, occurring in the 450 mg group at Day 141. In the MAD phase, no treatment-emergent ADAs were detected in participants treated with CM512 during the study. No pre-existing ADAs or treatment-boosted ADAs were observed across either phase, and no ADA responses were detected in the placebo group. Given the low incidence of ADA, the impact of immunogenicity on systemic exposure, PD, or safety could not be meaningfully assessed.

## 4 Discussion

The advent of biologics has transformed the treatment paradigm for type 2 inflammatory diseases. Advances in understanding the underlying pathological mechanisms have further facilitated the identification of novel therapeutic targets. Among these, IL-13 and TSLP have emerged as key pathogenic drivers that garnered significant attention in recent years. Monoclonal antibodies targeting these cytokines have shown clinical efficacy and received approval for the treatment of atopic dermatitis and asthma, while additional type 2 inflammatory indications are under active investigation (22). Bispecific antibodies that simultaneously target IL-13 and TSLP have been developed, with two agents, lunsekimig and CM512, currently in clinical evaluation. Encouraging phase 1 results with lunsekimig in healthy participants have supported the therapeutic potential of this dual-targeting strategy (20). In this first-in-human phase 1 study, CM512 was well tolerated in healthy participants and exhibited linear PK and low immunogenicity. Moreover, CM512 induced marked reductions in type 2 inflammatory biomarkers, supporting its clinical investigation in type 2 inflammatory diseases.

Both single and multiple ascending doses of subcutaneous CM512 demonstrated favorable safety and tolerability in healthy participants. The incidence of TEAEs was generally similar between CM512 and placebo groups across both the SAD and MAD phases. All reported treatment-related TEAEs were Grade 1 or 2 in severity, with no serious adverse events or discontinuations due to TEAEs. Hypertriglyceridemia was the most frequently reported TEAE in the pooled CM512 group (SAD: 28.1%, MAD: 25.0%). However, this event was also observed in the placebo group and did not show a dose−dependent trend, suggesting that it may be related to non−drug factors such as individual metabolic variability (23). Notably, lunsekimig has also shown a favorable safety and tolerability profile in phase 1 trial, with commonly reported TEAEs including COVID−19, nasopharyngitis, and headache (20), further supporting the safety potential of this dual−target pathway. Larger and longer−term studies in patient populations are warranted to fully characterize the long−term safety profile of CM512.

CM512 exhibited linear PK, with both peak and total exposure (C_max_, AUC_0-t_, and AUC_inf_) increasing proportionally with dose across the 150–1200 mg range. After a single subcutaneous administration of CM512 in healthy participants, absorption was observed to be slow, with notable interindividual variability. The median T_max_ ranged from approximately 6 to 8.5 days. CM512 exhibited a long and dose-independent mean t_1/2z_ (59–74 days), which is notably longer than those reported for other antibodies targeting related pathways, such as lunsekimig (anti-TSLP/IL-13, 9.4-10.8 days), tezepelumab (anti-TSLP, 24–26 days), and lebrikizumab (anti-IL-13, 24.5 days) (20, 24, 25). This extended half-life, combined with dual-target inhibition of both upstream (TSLP) and downstream (IL-13) pathways, may offer differentiated dosing convenience and broader suppression of type 2 inflammation. Following multiple dosing, mean serum concentrations at steady state were higher after the last administration than the first, and systemic exposure increased approximately in proportion to dose, with a mean accumulation ratio of 2.50-3.17.

Compared with placebo, subcutaneous administration of CM512 as single or multiple doses in healthy participants demonstrated effective target engagement with its direct targets at the mechanistic level, as shown by sustained reductions in free TSLP and IL-13 from baseline. Although post-dose measurements for both cytokines frequently fell below the LLOQ/LOD, precluding precise quantification of the extent of reduction, the consistent pattern of undetectable levels after treatment provides strong supportive evidence for the intended pharmacodynamic mechanism of dual TSLP/IL-13 blockade. This finding contrasts with results from a phase 1 study of lunsekimig (20), in which quantification of total cytokine levels (free plus drug-bound) revealed post-dose increases in TSLP and IL-13, attributable to delayed clearance of accumulated drug-target complexes. Together, these methodologically complementary studies reinforce that dual-target blockade of TSLP and IL-13 successfully achieves its expected pharmacodynamic engagement. CM512 treatment also showed broad suppression of type 2 inflammation, as indicated by significant decreases in blood EOS, IgE, and TARC levels. No clear dose-dependent trends were observed for these changes in downstream biomarkers.

This study represents the first-in-human investigation of single and multiple doses of subcutaneous CM512. Owing to its exploratory nature, several limitations should be acknowledged, including the small sample size and short treatment duration. Additionally, as the overall assessment was conducted in healthy volunteers, confirmatory evaluation in patients with type 2 inflammatory diseases, such as atopic dermatitis (the target population of the ongoing Phase I program), remains warranted. Furthermore, as an initial Phase I study, the study population consisted predominantly of Han Chinese participants, and confirmatory studies in more ethnically diverse populations are needed in later-stage development to establish the generalizability of these findings. Nevertheless, the study provides a preliminary characterization of the safety, PK, PD, and immunogenicity of CM512 in healthy participants.

## 5 Conclusions

In summary, single and multiple subcutaneous doses of CM512 were well tolerated in healthy participants. CM512 demonstrated linear PK with an extended half-life and low immunogenicity. Treatment with CM512 led to significant reductions in blood EOS, IgE, and TARC compared with placebo, supporting its potential as a therapeutic agent for type 2 inflammatory diseases. The sustained decreases in circulating free TSLP and IL-13 further confirmed effective target engagement. These findings provide preliminary evidence supporting the continued clinical development of CM512 in patient populations.

## Data Availability

The raw data supporting the conclusions of this article will be made available by the authors, without undue reservation.
