# Supplementary figures and images for: First-in-human, phase 1 study of CM512, a TSLP/IL-13 bispecific antibody, in healthy volunteers: safety, tolerability, pharmacokinetics, pharmacodynamics, and immunogenicity
Source: Front Immunol. 2026 May 28;17:1811041. doi: 10.3389/fimmu.2026.1811041 (PMC13254703; doi:10.3389/fimmu.2026.1811041)

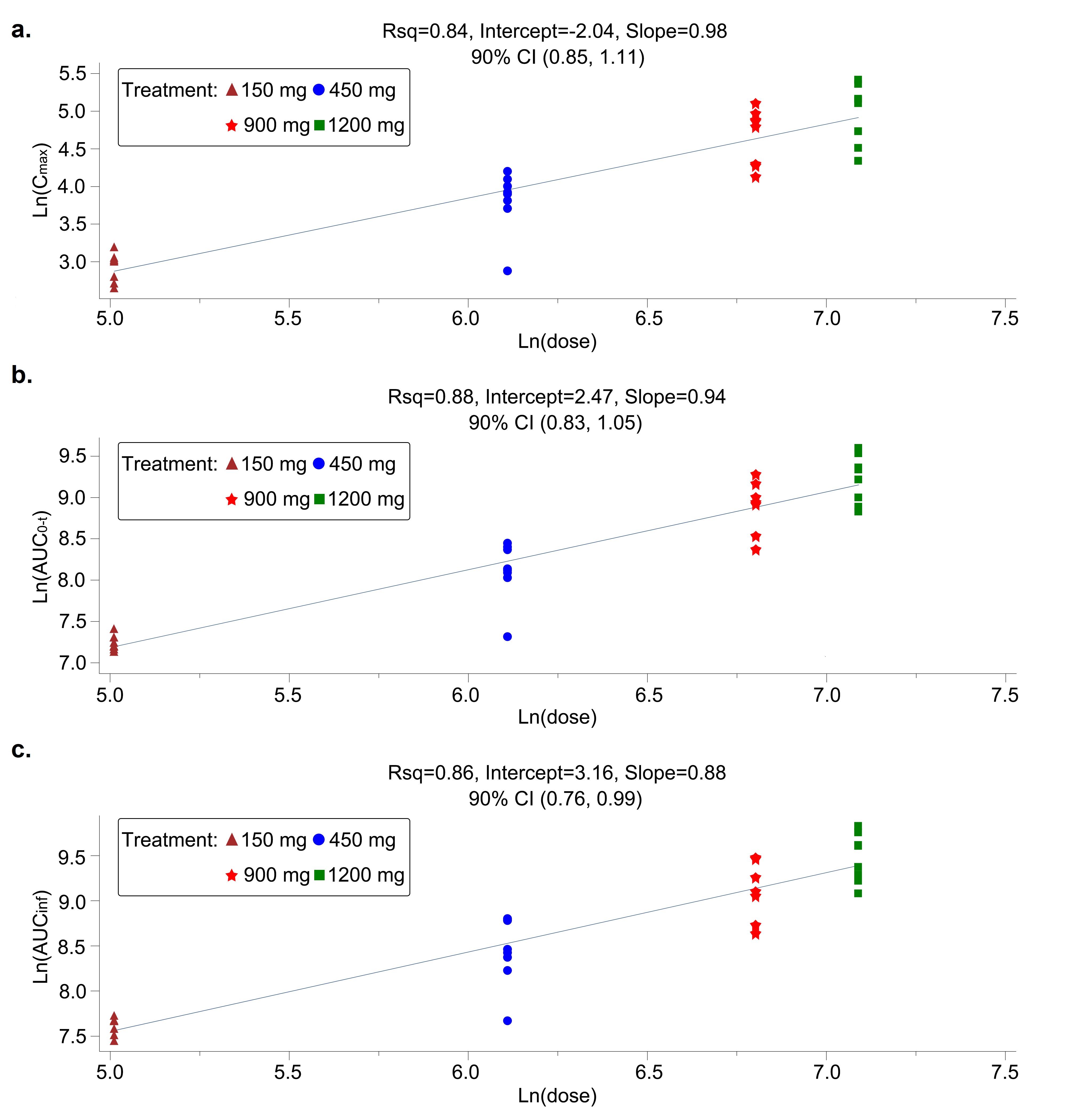

Supplement: Supplementary file 2 [file Image2.jpeg]
