# Supplementary material for: First-in-human, phase 1 study of CM512, a TSLP/IL-13 bispecific antibody, in healthy volunteers: safety, tolerability, pharmacokinetics, pharmacodynamics, and immunogenicity
Source: Front Immunol. 2026 May 28;17:1811041. doi: 10.3389/fimmu.2026.1811041 (PMC13254703; doi:10.3389/fimmu.2026.1811041)
Supplement: Supplementary file 3 [file Table1.docx]

## **Supplementary Methods.**

**1 Inclusion and Exclusion Criteria**

Participants who met all of the following inclusion criteria and none of the exclusion criteria were enrolled:

**1.1 Inclusion Criteria:**

1. Able to understand and comply with study-related procedures, and voluntarily sign the Informed Consent Form (ICF).
2. Aged between 18 and 45 years (inclusive) at the time of signing the ICF, regardless of sex.
3. Assessed by the investigators as being in good general health status based on medical history, physical examination, vital signs, and laboratory test results at screening.
4. Normal or abnormal but clinically non-significant ECG and chest X-ray findings at screening.
5. Body mass index (BMI) between 18.0 and 26.0 kg/m² (inclusive).

**1.2 Exclusion Criteria:**

1. History of allergy to anti-IL-13 or anti-TSLP monoclonal antibodies, or history of severe drug or food allergies, or judged by the investigator as potentially allergic to the investigational medicinal product.
2. Participation in any clinical trial of drugs or medical devices within 3 months prior to screening.
3. History of severe infection, severe trauma, or major surgery within 3 months prior to screening.
4. Blood loss exceeding 400 mL due to blood donation or other reasons within 3 months prior to screening.
5. Systemic infectious disease requiring treatment with antibiotics, antivirals, antiparasitics, antiprotozoals, or antifungals within 4 weeks prior to screening.
6. Administration of a live attenuated vaccine within 4 weeks prior to randomization or planned during the study; administration of an inactivated or recombinant vaccine within 1 week prior to randomization.
7. Positive for any of the following: Hepatitis B surface antigen (HBsAg), Hepatitis B core antibody (HBcAb), Hepatitis C virus antibody (HCV-Ab), Human Immunodeficiency Virus antibody (HIV-Ab), or Treponema pallidum-specific antibody (TP-Ab).

Note: Participants positive for HBcAb but positive for Hepatitis B surface antibody (HBsAb) may be enrolled.

1. Active *Mycobacterium tuberculosis* infection (i.e., tuberculosis) at screening or in medical history.
2. Positive drug abuse screen or positive alcohol breath test.
3. Average daily smoking of >5 cigarettes within 3 months prior to screening, or inability to discontinue use of any tobacco products during the trial.
4. Weekly average alcohol intake >14 units (1 unit = 360 mL of beer, or 45 mL of 40% spirits, or 150 mL of wine) within 3 months prior to screening, or inability to abstain from alcohol during the trial.
5. Habitual consumption of >1 L daily of strong tea, coffee, and/or caffeine-containing beverages within 3 months prior to screening; or consumption of chocolate or any food/beverage containing caffeine, theophylline, theobromine, or alcohol within 24 hours prior to investigational medicinal product administration.
6. Use of any prescription drugs, over-the-counter medications, vitamin products, proprietary Chinese medicines, any Chinese herbal products, or any non-biological investigational agents within 4 weeks prior to screening.
7. History or presence of any chronic or severe disease involving the musculoskeletal, neuropsychiatric, endocrine, circulatory, respiratory, digestive, urinary, reproductive systems, or other conditions deemed by the investigator as unsuitable for clinical trial participation.
8. Any condition at the intended injection site (e.g., skin depression, induration, scar, inflammation, edema, ulcer, infection, bleeding) judged by the investigator as unsuitable for subcutaneous injection.
9. Requirement or plan to engage in strenuous physical activity or exercise during the study period.
10. For participants of childbearing potential or male participants, planning pregnancy or sperm donation from ICF signing until 5 months after investigational medicinal product administration, or unwilling to use highly effective contraception from ICF signing until 5 months after investigational medicinal product administration.
11. Pregnancy, lactation, or abnormal and clinically significant human chorionic gonadotropin (HCG) level at screening.
12. Judged by the investigator as unsuitable for clinical trial participation or unable to complete the study for other reasons.

**2 Rationale for Dose Selection**

The starting dose for CM512 was determined based on the National Medical Products Administration (NMPA) guideline "Guideline for Estimating the Maximum Recommended Starting Dose for Initial Clinical Trials of Drugs in Adult Healthy Volunteers" and the US Food and Drug Administration guidance "Guidance for Industry: Estimating the Maximum Safe Starting Dose in Initial Clinical Trials for Therapeutics in Adult Healthy Volunteers". Considerations were as follows:

1. **Estimation Based on No Observed Adverse Effect Level (NOAEL)**
   CM512, a bispecific antibody, binds with high affinity to both TSLP and IL-13 in humans and cynomolgus monkeys with comparable affinity, indicating the cynomolgus monkey is a relevant species for preclinical evaluation.
   According to the repeat-dose toxicity study in cynomolgus monkeys, the NOAEL was 150 mg/kg. Using a safety factor of 10 and a body surface area conversion factor of 0.32 from cynomolgus monkey to human, and assuming a standard human weight of 60 kg, the Maximum Recommended Starting Dose (MRSD) was calculated to be 288 mg (equivalent to 4.8 mg/kg).
2. **Estimation Based on Minimal Anticipated Biological Effect Level (MABEL)**
   CM512 is a bispecific antibody targeting TSLP and IL-13, which inhibits immune cell proliferation and inflammatory cytokine release induced by TSLP and IL-13 via high-affinity binding. Therefore, CM512 is not an immune agonist, and the MABEL approach is not applicable for estimating its starting dose.
3. **Estimation Based on Preclinical In Vivo Pharmacologically Active Dose (PAD)**
   In the MC903-induced B-hTSLP/hTSLPR mouse dermatitis model, partial biological activity (e.g., inhibition of ear thickness increase, improvement of ear lesions, reduction of serum total IgE levels, suppression of ear tissue inflammation) was observed after multiple subcutaneous administrations of CM512 at 30 mg/kg. Using a body surface area conversion factor of 0.08 from mouse to human and a standard human weight of 60 kg, the corresponding human dose was calculated to be 144 mg (equivalent to 2.4 mg/kg). Considering the drug specification of 300 mg (2 mL) per vial, the clinical MRSD was adjusted to 150 mg. This dose is approximately half of the MRSD estimated based on NOAEL, providing a better safety margin.

In summary, based on the comprehensive preclinical data, the clinical starting dose for subcutaneous administration of CM512 was determined to be 150 mg. Following the principle of decreasing dose escalation increments, the planned dose escalation levels are 150 mg, 450 mg, 900 mg, and 1200 mg. The highest planned dose of 1200 mg maintains an approximately 8-fold safety margin relative to the NOAEL in cynomolgus monkeys (150 mg/kg).

# **Supplementary tables**

## **Table S1.** Summary of TEAEs for the SAD phase.

| **TEAEs by SOC/PT** | **Pooled placebo**  **(*n*=8)** | **Pooled CM512**  **(*n*=32)** | **CM512 150 mg**  **(*n*=8)** | **CM512 450 mg**  **(*n*=8)** | **CM512 900 mg**  **(*n*=8)** | **CM512 1200 mg**  **(*n*=8)** |
| --- | --- | --- | --- | --- | --- | --- |
| **Metabolism and nutrition disorders** | **2 (25.0)** | **13 (40.6)** | **3 (37.5)** | **4 (50.0)** | **4 (50.0)** | **2 (25.0)** |
| Hypertriglyceridaemia | 1 (12.5) | 9 (28.1) | 3 (37.5) | 1 (12.5) | 4 (50.0) | 1 (12.5) |
| Hypercholesterolaemia | 0 | 2 (6.3) | 0 | 1 (12.5) | 1 (12.5) | 0 |
| Hyperuricaemia | 1 (12.5) | 2 (6.3) | 0 | 1 (12.5) | 0 | 1 (12.5) |
| Hypoglycaemia | 0 | 1 (3.1) | 0 | 1 (12.5) | 0 | 0 |
| **Investigations** | **3 (37.5)** | **12 (37.5)** | **1 (12.5)** | **4 (50.0)** | **5 (62.5)** | **2 (25.0)** |
| Alanine aminotransferase increased | 1 (12.5) | 5 (15.6) | 0 | 3 (37.5) | 2 (25.0) | 0 |
| White blood cells urine positive | 0 | 3 (9.4) | 0 | 1 (12.5) | 1 (12.5) | 1 (12.5) |
| Aspartate aminotransferase increased | 0 | 3 (9.4) | 0 | 1 (12.5) | 1 (12.5) | 1 (12.5) |
| Gamma-glutamyltransferase increased | 0 | 2 (6.3) | 0 | 1 ( 12.5) | 1 (12.5) | 0 |
| White blood cell count increased | 1 (12.5) | 2 (6.3) | 1 (12.5) | 0 | 1 (12.5) | 0 |
| Neutrophil count increased | 1 (12.5) | 2 (6.3) | 1 (12.5) | 0 | 1 (12.5) | 0 |
| Blood creatine phosphokinase increased | 0 | 1 (3.1) | 0 | 0 | 0 | 1 (12.5) |
| Blood fibrinogen decreased | 1 (12.5) | 1 (3.1) | 0 | 0 | 1 (12.5) | 0 |
| **Infections and infestations** | **1 (12.5)** | **4 (12.5)** | **0** | **1 (12.5)** | **2 (25.0)** | **1 (12.5)** |
| Upper respiratory tract infection | 0 | 3 (9.4) | 0 | 0 | 2 (25.0) | 1 (12.5) |
| Urinary tract infection | 0 | 1 (3.1) | 0 | 1 (12.5) | 0 | 0 |
| Pulpitis dental | 1 (12.5) | 0 | 0 | 0 | 0 | 0 |
| **Nervous system disorders** | **0** | **2 (6.3)** | **0** | **2 (25.0)** | **0** | **0** |
| Headache | 0 | 1 (3.1) | 0 | 1 (12.5) | 0 | 0 |
| Dizziness | 0 | 1 (3.1) | 0 | 1 (12.5) | 0 | 0 |
| **General disorders and administration site conditions** | **0** | **2 (6.3)** | **0** | **2 (25.0)** | **0** | **0** |
| Pyrexia | 0 | 1 (3.1) | 0 | 1 (12.5) | 0 | 0 |
| Injection site haemorrhage | 0 | 1 (3.1) | 0 | 1 (12.5) | 0 | 0 |
| **Blood and lymphatic system disorders** | **0** | **2 (6.3)** | **0** | **1 (12.5)** | **1 (12.5)** | **0** |
| Anaemia | 0 | 2 (6.3) | 0 | 1 (12.5) | 1 (12.5) | 0 |
| **Skin and subcutaneous tissue disorders** | **1 (12.5)** | **1 (3.1)** | **0** | **0** | **1 (12.5)** | **0** |
| Pruritus | 0 | 1 (3.1) | 0 | 0 | 1 (12.5) | 0 |
| Urticaria | 1 (12.5) | 0 | 0 | 0 | 0 | 0 |
| **Cardiac disorders** | **0** | **1 (3.1)** | **0** | **1 (12.5)** | **0** | **0** |
| Sinus bradycardia | 0 | 1 (3.1) | 0 | 1 (12.5) | 0 | 0 |
| **Psychiatric disorders** | **1 (12.5)** | **0** | **0** | **0** | **0** | **0** |
| Insomnia | 1 (12.5) | 0 | 0 | 0 | 0 | 0 |

Note: PT, Preferred term; SAD, single ascending dose; SOC, System organ class; TEAEs, treatment emergent adverse events.

## **Table S2.** Summary list of TEAEs for the MAD phase.

| **TEAEs by SOC/PT** | **Pooled placebo**  **(*n*=8)** | **Pooled CM512**  **(*n*=16)** | **CM512 150mg Q2W**  **(*n*=8)** | **CM512 600mg Q2W**  **(*n*=8)** |
| --- | --- | --- | --- | --- |
| **Metabolism and nutrition disorders** | **2 (25.0)** | **5 (31.3)** | **3 (37.5)** | **2 (25.0)** |
| Hypertriglyceridaemia | 2 (25.0) | 4 (25.0) | 2 (25.0) | 2 (25.0) |
| Hypoglycaemia | 0 | 1 (6.3) | 1 (12.5) | 0 |
| **Investigations** | **3 (37.5)** | **5 (31.3)** | **3 (37.5)** | **2 (25.0)** |
| White blood cell count increased | 0 | 2 (12.5) | 1 (12.5) | 1 (12.5) |
| Alanine aminotransferase increased | 1 (12.5) | 2 (12.5) | 1 (12.5) | 1 (12.5) |
| Blood creatine phosphokinase increased | 2 (25.0) | 2 (12.5) | 1 (12.5) | 1 (12.5) |
| Lymphocyte count increased | 0 | 1 (6.3) | 0 | 1 (12.5) |
| White blood cells urine positive | 0 | 1 (6.3) | 1 (12.5) | 0 |
| Urobilinogen urine increased | 0 | 1 (6.3) | 1 (12.5) | 0 |
| Blood fibrinogen decreased | 0 | 1 (6.3) | 0 | 1 (12.5) |
| Neutrophil count increased | 0 | 1 (6.3) | 1 (12.5) | 0 |
| Aspartate aminotransferase increased | 2 (25.0) | 0 | 0 | 0 |
| **Infections and infestations** | **1 (12.5)** | **4 (25.0)** | **3 (37.5)** | **1 (12.5)** |
| Upper respiratory tract infection | 1 (12.5) | 2 (12.5) | 2 (25.0) | 0 |
| Pericoronitis | 0 | 1 (6.3) | 1 (12.5) | 0 |
| Hordeolum | 0 | 1 (6.3) | 0 | 1 (12.5) |
| **Skin and subcutaneous tissue disorders** | **0** | **1 (6.3)** | **1 (12.5)** | **0** |
| Urticaria | 0 | 1 (6.3) | 1 (12.5) | 0 |
| **General disorders and administration site conditions** | **0** | **1 (6.3)** | **0** | **1 (12.5)** |
| Injection site erythema | 0 | 1 (6.3) | 0 | 1 (12.5) |

Note: PT, Preferred term; MAD, multiple ascending dose; SOC, System organ class; TEAEs, treatment emergent adverse events.

**Supplementary figures:**

**Figure S1. Participant flow diagram of the SAD and MAD phases of the clinical trial.** **(a)** SAD phase; **(b)** MAD phase. FAS, full analysis set; IMGS, immunogenicity set; MAD, multiple ascending dose; PDS, pharmacodynamic set; PKCS, pharmacokinetic concentration set; PKPS, pharmacokinetic parameter set; SAD, single ascending dose; SS, safety set.

**Figure S2.** Dose proportionality of CM512 pharmacokinetic parameters in healthy participants following a single subcutaneous dose in the SAD phase. **(a)**: C_max_; **(b)**: AUC_0–t_; **(c)**: AUC_inf_. AUC_0-t_, area under the concentration-time curve from time zero to the last quantifiable concentration; AUC_inf_, area under the concentration-time curve from time zero extrapolated to infinity; C_max_, maximum concentration; SAD, single ascending dose.
